# Progresses and Prospects of Neuroprotective Agents-Loaded Nanoparticles and Biomimetic Material in Ischemic Stroke

**DOI:** 10.3389/fncel.2022.868323

**Published:** 2022-04-11

**Authors:** Junfa Chen, Jing Jin, Kaiqiang Li, Lin Shi, Xuehua Wen, Fuquan Fang

**Affiliations:** ^1^Center for Rehabilitation Medicine, Department of Radiology, Zhejiang Provincial People’s Hospital (Affiliated People’s Hospital, Hangzhou Medical College), Hangzhou, China; ^2^Laboratory Medicine Center, Zhejiang Center for Clinical Laboratory, Zhejiang Provincial People’s Hospital (Affiliated People’s Hospital, Hangzhou Medical College), Hangzhou, China; ^3^Laboratory Medicine Center, Department of Transfusion Medicine, Zhejiang Provincial People’s Hospital (Affiliated People’s Hospital, Hangzhou Medical College), Hangzhou, China; ^4^Department of Anesthesiology, First Affiliated Hospital, Zhejiang University School of Medicine, Hangzhou, China

**Keywords:** ischemic stroke, neuroprotection, nanoparticles, biomimetic material, blood-brain barrier

## Abstract

Ischemic stroke remains the leading cause of death and disability, while the main mechanisms of dominant neurological damage in stroke contain excitotoxicity, oxidative stress, and inflammation. The clinical application of many neuroprotective agents is limited mainly due to their inability to cross the blood-brain barrier (BBB), short half-life and low bioavailability. These disadvantages can be better eliminated/reduced by nanoparticle as the carrier of these drugs. This review expounded the currently hot researched nanomedicines from the perspective of the mechanism of ischemic stroke. In addition, this review describes the bionic nanomedicine delivery strategies containing cells, cell membrane vesicles and exosomes that can effectively avoid the risk of clearance by the reticuloendothelial system. The potential challenges and application prospect for clinical translation of these delivery platforms were also discussed.

## Introduction

Ischemic stroke is considered one of the most threatening neurological diseases, accounting for 85% of all stroke cases and mortality rate of ischemic stroke within 30 days has been estimated at around 15% in high-income countries (Feigin et al., 2014). An ischemic stroke occurs when any of the many arteries that supply the brain is blocked, resulting in reduced blood flow to the brain. This occlusion is followed by a lack of blood supply (ischemia) and a lack of oxygen (hypoxia) and nutrients to the brain (Ham and Raju, 2017). Subsequently, a cascade of biochemical reactions leads to neuronal cell death and neuroinflammation, ultimately leading to the loss of neural function. Even with reperfusion of ischemic tissue, hyper oxygenated blood will generate reactive oxygen species, resulting in oxidative damage (Carbone et al., 2015; Akbik et al., 2020). Because stroke location is determined by occluded blood vessels and often spans multiple functional areas, the resulting symptoms are often a combination of motor, cognitive, and psychiatric disorders.

Despite understanding of the pathophysiology of stroke is increasing, efficient treatment remains a major challenge in clinical. To date, the treatment strategy of ischemic stroke approved by the Food and Drug Administration (FDA) was the use of tissue plasminogen activator (tPA), while mechanical thrombectomy as well as specific catheters have also been approved recently (Pena et al., 2019). tPA was reported to have a dominant significant curative effect in small to moderate-sized strokes, but played no significant role in large-vessel occlusions (Lekoubou et al., 2017) and had narrow time window for thrombolysis (Shi et al., 2021), limiting their use in a significant number of patients. Although rapid reperfusion is necessary for restoration of brain metabolic activity, it is also associated with irreversible neurological damage (Mizuma et al., 2018). Therefore, the current goal is to develop neuroprotection strategies which prevent brain cells injury in both ischemia and reperfusion, as well as to extend the time window for thrombolytic treatment (Chamorro et al., 2016). However, for many neuroprotective agents, resistance to clinical application includes inability to cross the blood-brain barrier (BBB), short half-life and low bioavailability (Tam et al., 2014).

In the recent decades, the potential therapeutic ability of many natural and artificial polymers has come to light (Obermeyer et al., 2019; He et al., 2021a). Different biomaterials [e.g., nanoparticles (NPs), hydrogels, and nanotubes] that have biocompatibility with nervous tissue have been designed as drug delivery platforms, thus reducing the rapid degradation and decay of activity of compounds with poor half-lives, leading to an enhanced therapeutic outcome. To further enhance the delivery efficiency of these drug delivery system, NPs surface functionalization with specific ligands is also performed (Bao et al., 2018). In addition to potential for controlled drug release, several hydrogels and NPs possess the special ability to attenuate inflammation and oxidative stress induced by brain injury on their own (Ghuman et al., 2016; Nih et al., 2017).

In this review, we summarize the current neuroprotective strategies based on the pathological mechanism of ischemic stroke. Furthermore, we discuss the main and advanced biomaterials as delivery platforms of neuroprotective compounds for each neuroprotective strategy, as well as potential challenges for clinical translation of these delivery platforms.

## Mechanism of Neural Damage in Ischemic Stroke

**FIGURE 1:**
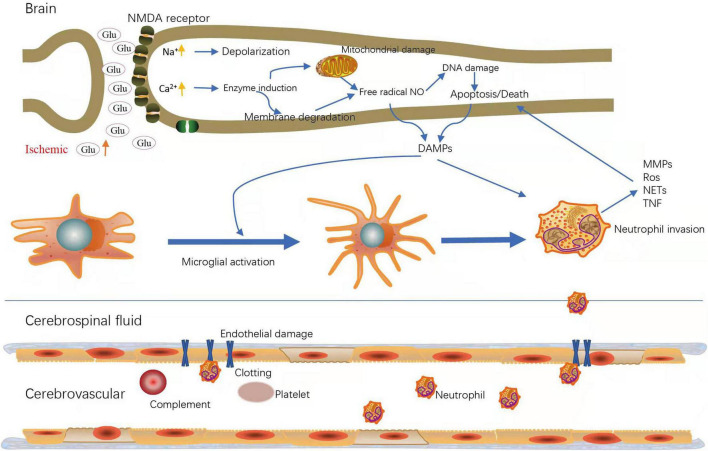
Major active cells and molecules during ischemic stroke. Upregulation of glutamate (glu) and increased NMDA (*N*-methyl-D-aspartate) receptors lead to intracellular calcium (Ca2+) and sodium (Na+) loading; Increased intracellular Ca2+ induce cell membrane degradation and mitochondrial damage, followed by apoptosis or death; DAMPs (Damage-associated molecular patterns) trigger microglial activation to release chemokines, which can induce neutrophil invasion; Ischemia leads to endothelial damage, resulting in increased vascular permeability and inflammatory cell migration. MMPs, matrix metalloproteinase; Ros, reactive oxygen species; NETs, neutrophil extracellular traps; TNF, Tumor Necrosis Factor.

Ischemic stroke contains multiple mechanisms, which superimpose and exert undesirable effects, leading to brain damage. Neurons in ischemic lesions lack sufficient blood and oxygen supply, which accelerates their necrosis and apoptosis, forming a core infarct area surrounded by penumbra. The brain damage caused by ischemic stroke is a dynamic process. In the acute phase, the insufficient oxygen and energy supply causes a series of stress responses, which up-regulate reactive oxygen free radicals (ROS) and cytokines and activate microglia and astrocytes. Subsequently, cytokines secreted by glial cells destroy the integrity of the blood brain barrier (BBB), and recruit peripheral neutrophils migrate into the brain parenchyma, which intensifies the inflammatory response, thus leading to brain edema, BBB damage and neuronal death. During the period of chronic recovery, macrophages migrate to the cerebral ischemic area to participate in the regeneration of neurons (Gronberg et al., 2013; Iadecola et al., 2020). In general, death executors include abnormal excitability, inflammation, oxidative stress, and spreading depolarization (Figure 1).

### Excitotoxicity

Excitotoxicity occurs after a stroke due to massive excitatory neurotransmitters uncontrolled released by injured neurons. The re-uptake of excitatory neurotransmitters was failed due to insufficient energy intake, leading to the accumulation of excitatory neurotransmitters in the synaptic. The impairment of ionic gradients triggers the depolarization of brain cells, taking the intracellular potassium depletion and influx of calcium and sodium as dominant feature, ultimately leading to cerebral edema. Massive increase of excitatory amino acid neurotransmitters in the extracellular space occurs parallelly to the changes in resting membrane potential, which include abundant glutamate (Lewerenz and Maher, 2015; Amantea and Bagetta, 2017). The excessive activation of glutamatergic receptors (NMDA, AMPA, and kainate) further promotes the influx of potassium and calcium. The activation of calcium-dependent pathways including the activation of NO synthase and proteases, producing the following pathological containing reaction oxidative stress, mitochondrial dysfunction, and modifications of gene expression and protein activation level, eventually result in cells necrosis and death (Kostandy, 2012). Excitotoxicity is mediated by uncontrolled release of neurotransmitters such as adenosine and glutamate, as well as an overload of intracellular calcium (Chamorro et al., 2016). Due to the self-evident important role of glutamate receptors in the evolution of ischemic stroke, Glutamate receptor antagonists and calcium channel blockers have been designed to prevent mitochondrial dysfunction, suppress excitotoxicity and exert neuroprotective effects.

### Oxidative Stress

Oxidative stress plays a vital role in the pathogenesis of ischemia-reperfusion process (Carbone et al., 2015; Cheng et al., 2017) and has deleterious effect in the pathogenesis of post-stroke neurological dysfunction. After ischemia/reperfusion, adequate aerobic respiration is blocked for the dysfunction of mitochondrial, resulting in overproduction of nitrogen and oxygen species (RNOS), exceeding the capability of RNOS clearance. Furthermore, reactive oxygen producing enzymes and reduction of antioxidant enzymes is activated. The breakdown of oxidative balance causes oxidative stress, which may take the form of nitration or oxidation of various amino acid residues. Excessive ROS will cause neuronal autophagy and apoptosis through multiple pathological process including lipid peroxidation, protein oxidation and denaturation, protein aggregation, DNA fragmentation. By different matrix metalloproteinase activated by ROS, free radicals released from intracellular organelles arouse damage to the cytoskeleton, cerebral edema, and the destroy of BBB (Moro et al., 2005; Chamorro et al., 2016).

### Inflammation

Inflammation is a component of the pathophysiology of the brain in stroke, contributing to neuropil damage (Lambertsen et al., 2019). In the acute phase, the activated intracerebral immune cells-microglia accompany with DAMPs released from apoptotic and necrotic cells participate in the immune response (Liesz et al., 2015; Gulke et al., 2018). The “classically activated” M1-phenotype microglia can release pro-inflammatory cytokines (TNFα, IL-1α/β) and produce cytotoxic factors to provoke inflammatory response. Besides, cytokines and cytotoxic factors destroy the tight junctions of the BBB, eventually causing the breakdown of the BBB (Anrather and Iadecola, 2016). For the transient disruption of the BBB, many peripheral immune cells will be recruited to the lesion site, and further release overwhelming cytokines, chemokines and other cytotoxic mediators, causing the inflammatory cascade reaction (Anselmo and Mitragotri, 2014; Cheng Z. et al., 2019). Meanwhile, peripheral immune cells infiltrate cause neuronal cell death, followed by more immune cells attracted by the death into the lesion (Anrather and Iadecola, 2016). The strategies to suppress the activation of the immune system have mostly failed in the clinic (Fu et al., 2015; Mizuma and Yenari, 2017).

## The Nanoparticles as Drug Carriers in Ischemic Stroke

**FIGURE 2:**
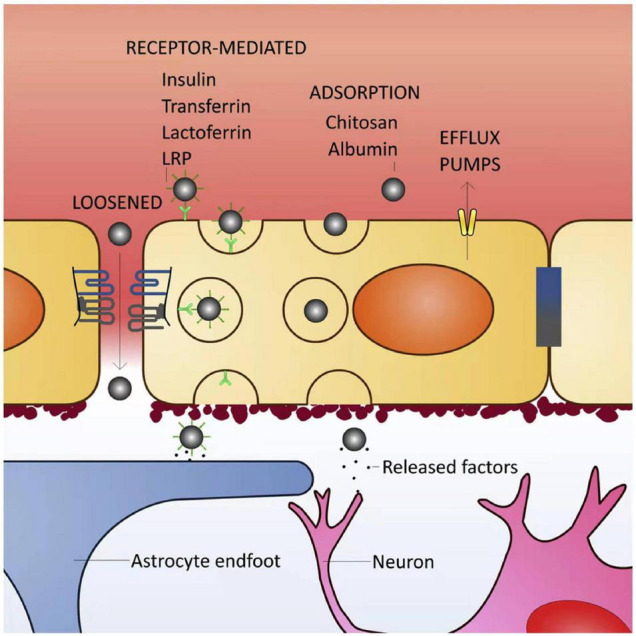
Blood–brain barrier (BBB) transport mechanisms for brain delivery of nanoparticles (NPs). The BBB is highly selective and has specific transport mechanisms allowing a close control of molecules/cells that enter the brain parenchyma. Loosened tight junctions (TJs) allow the cross of NPs through the BBB, either by the presence of a surfactant in NPs able to disrupt the TJs or by BBB impairment due to pathological conditions. Receptor-mediated transcytosis is the most common type of transport for NP entry into the brain. NPs can be functionalized with different types of ligands (such as insulin, transferrin, lactoferrin or antibodies against some endothelial receptors), or surfactants like polysorbate 80 [that adsorbs plasma proteins, namely apolipoprotein E enabling their binding to the lipoprotein receptor-related proteins (LRPs)]. The interaction between NP ligands and respective receptors in the endothelial cell (luminal side) surface triggers plasma membrane invaginations followed by pinch free forming vesicles, which facilitates the release of the NPs in the opposite site of the membrane (parenchymal side). NPs coated with molecules such as albumin or chitosan can cross the BBB by adsorptive transcytosis. Efflux pumps may reduce the amount of NPs retained in brain parenchyma (Saraiva et al., 2016).

In recent years, the rapid development of NPs has garnered considerable interest in neural tissue reconstruction as a promising treatment strategy for stroke repair. NPs could be natural or synthetic 3D polymer networks varied from 1 to 1000 nm in size. The NPs can transport drugs by adsorbing, entrapping, or bounding covalently to them. These carriers are used to act as a drug delivery vehicle releasing neuroprotective agents in a spatiotemporally controlled manner or provide a suitable microenvironment for injured cells to restore biological function (Modo et al., 2013, 2018; Figure 2).

When NPs carry the drug up to the destination area, they can control the rate of release. The control of release rate is based on the signals of initiate migration and cell invasion that are induced by biomaterials (Massensini et al., 2015). The carrier can act as a temporary protective barrier for medicine against the unbalanced microenvironment in ischemic tissue, which increase the effectiveness of the treatment in the target. It is noteworthy that certain nano drug delivery system can not only load drugs to treat ischemic stroke, but also utilized as imaging probes for tracking and imaging (Agulla et al., 2013). Implantations of these scaffolds at an injury site may not only reduce stroke mortality but restore lost neurological functions through the regeneration of neural tissue. In the following, the currently widely used or novel NPs will be described based on the drug brain protection mechanism.

### Anti-inflammatory Drug-Loaded Nanoparticles

#### Lipid Nanoparticles

Lipid NPs have been introduced with the objective to not only increase delivery load but also decrease expulsion of drug (Joshi et al., 2019). Liposomes are biodegradable and biocompatible, with high loading capacity, and are able to cross the BBB. The injected liposomes were allowed to leak into the brain parenchyma, and then these liposomes gradually accumulated in the ischemic region during the acute phase of cerebral ischemia, due to the enhanced permeability and retention effect. Liposomes act as carriers of the neuroprotective agents FK506 (Fukuta et al., 2015) and Cyclosporin A (Partoazar et al., 2017), enabling them to pass through the blood-brain barrier more easily and function more efficiently. After the infusion of these two protective agents, infarct size was reduced, leukocyte infiltration was inhibited and the expression of TNF-α was reduced. Xenon (Xe), a noble gas, was reported to have promising neuroprotective properties with no adverse side effects (Altschuler, 2001). The Xe delivered by echogenic liposomes was proved to reduced bleeding, reduced apoptotic neuronal death, and neutrophil infiltration (Miao et al., 2018), as shown in Table 1.

**TABLE 1:** Summary of targeted delivery of anti-inflammatory agents with nanomedicines.

Type of NPs	NPs	Agent	Mechanism of neuroprotection	Research species	References
Liposomes	DSPC, DPPC, DSPE-PEG 2000	FK506	Reduce infarct size, inhibited leukocyte infiltration and reduced the expression of TNF-α	Animal	Fukuta et al., 2015
	PS, DOPE, DSPE-PEG 2000	Cyclosporin A	Recover the infarct size, the brain edema, and the neurological activities; inhibit the inflammation responses including MPO activity and TNF-α level	Animal	Partoazar et al., 2017
	DPPC, Egg phosphocholine, PEG2000, DPPG, cholesterol	Xenon	Reduced apoptotic neuronal death and decreased mortality	Animal	Miao et al., 2018
Polymeric NPs	poly (ethylene glycol)-block-poly (D, L-lactide)	C3 siRNA	Decrease C3 expression in microglia and ischemic brain tissue; reduce the number of infiltrating inflammatory cells and the concentration of pro-inflammatory factors	Animal	Wang et al., 2018
	Poly (ethylene glycol)-b-poly(D,L-lactide)	Curcumin	Inhibited the increase in MMP-9; maintained BBB Integrity; reduced the number of activated M1 microglia and weakened the increase in TNF-α and IL-1β	Animal	Wang et al., 2019
	Unknown	miR-195	Anti-apoptosis for injured neural cells by directly suppressing Sema3A/Cdc42/JNK signaling; neural regeneration by promoting neural stem cell proliferation and migration; anti-inflammation by directly blocking the NF-kB pathway; improvement of endothelial functions	Animal	Cheng H. Y. et al., 2019
Inorganic NPs	Anti-transferrin receptor monoclonal Antibody (OX26)-PEGylated Se nanoparticles (OX26-PEG-Se NPs)	Se	Inhibit activity of jak2/stat3 signaling pathways and reduce the transcription level of inflammation-related factor Adamts1	Animal	Amani et al., 2019
	PLGA functionalized magnetic Fe3O4 nanoparticle (MNP)	Dexamethasone	Cross through the BBB; efficient drug loading rate; control releasing efficiency of the NPs	Animal	Lu et al., 2021
Carbon-Based NPs	Allotropic variation of carbon atom	Fullerenol	Reduce infarct volume; reduce the transcription of IL-6 and MMP-9 to protect BBB integrity; relieve brain edema after cerebral ischemia-reperfusion injury	Animal	Sarami Foroshani et al., 2018

*NPs, nanoparticles; TNF-α, Tumor necrosis factor-alpha; DSPC, Distearoylphosphatidylcholine; DPPC, dipalmitoyl-phosphatidylcholine; PS, phosphatidylserine; DOPE, Dioleoyl phosphatidylethanolamine; DSPE-PEG 2000, Distearoylphosphoethanolamine-polyethyleneglycol-2000; DPPG, 1,2-dipalmitoyl-sn-glycero-3-phospho.*

#### Polymeric Nanoparticles

**FIGURE 3:**
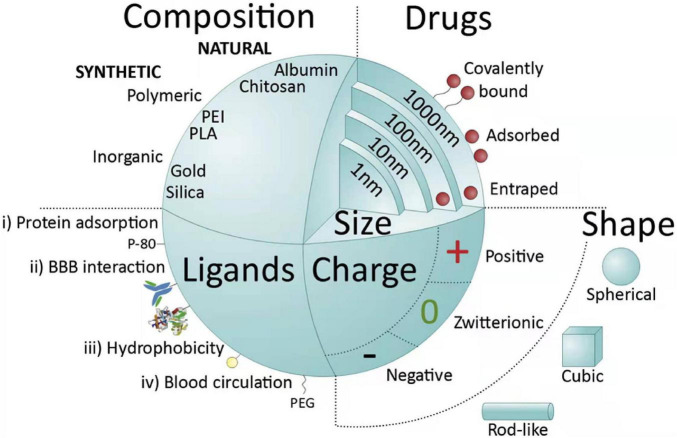
Main nanoparticle (NP) features influencing systemic delivery and blood brain barrier (BBB) passage. NPs can be classified into natural, when molecules such as proteins (albumin), polysaccharides, chitosan, among others are used, or synthetic. Synthetic NPs can be made of very common polymers such as poly(lactic-co-glycolic acid) (PLGA), poly(ethylenimine) (PEI), polyesters [poly(lactic acid)] (PLA), or from inorganic agents like gold, silica or alumina. NPs can vary in their size (1–1000 nm) and are able to deliver drugs into cells by entrapping, adsorbing or covalently bounding them. NPs can assume different shapes (spherical, cubic, and rod-like) and charges (negative, zwitterionic, and positive); negatively charged spheres are widely used in intravenous applications. Another important feature of NPs is the possibility of functionalization with different types of ligands. Ligands are distributed into four major categories: (i) capable of mediating protein adsorption [e.g., poly(sorbate) 80 (P-80)]; (ii) able to interact directly with the BBB (e.g., transferrin proteins, antibody or peptides); (iii) capable of increasing hydrophobicity (e.g., amphiphilic peptides); and (iv) able to improve blood circulation [e.g., poly(ethylene glycol) (PEG)] (Saraiva et al., 2016).

Polymeric NPs, in particular, are a promising choice as drug delivery platform for central nervous system targeting, due to their tunable architecture (10 to 1000 nm), non-toxicity, biocompatibility, and controllable drug release (Kreuter et al., 2002). Polymers used to synthesis NPs include both synthetic polymers and natural polymers. The common synthetic polymers include poly-n-butylcyanoacrylate (PBCA), polyesters lactic acid (PLA), and poly lactic-co-glycolic acid (PLGA), while the natural polymers contains polysaccharides, amino acids, and proteins (Saraiva et al., 2016). Synthetic NPs can be reproducibly synthesized with specific functional groups, and their size, stability, shape is controlled (Khan et al., 2018), as show in Figure 3. A copolymer of polyethylene glycol (PEG)-PLA could effectively deliver C3-siRNA and curcumin into ischemic penumbra across the blood-brain barrier (BBB) and significantly decrease C3 expression and inhibit M1-microglial activation (Wang et al., 2018). NP-curcumin can also reduce the infarct size and improved function recovery (Wang et al., 2019). RNA therapy has broad prospects and can prevent or treat complex diseases in many fields. miR-195 was proved to possess the potential to become a new drug to treat acute ischemic stroke for it can anti-inflammation by directly blocking the NF-kB pathway in both cell and animal models. The reduction of injured brain volume by treatment of miR-195 could be up to 45% in stroke rats through multiple mechanisms including antiapoptotic and anti-inflammatory pathways (Cheng H. Y. et al., 2019).

#### Inorganic Nanoparticles

Inorganic materials, such as silica and iron oxide, are employed to produce inorganic NPs, with both diagnostic and therapeutic functions, for their ability to be tracked by magnetic resonance imaging (MRI) (Saraiva et al., 2016). Previous research has confirmed Se take part in modulation of neurogenesis and Se administration contributes to mitochondrial dynamics after focal cerebral ischemia (Nissen-Druey, 1989). Anti-transferrin receptor antibody was synthesized with Se-NPs. The therapeutic effect of Se NPs was significantly effective in a murine stroke model because the activity of jak2/stat3 signaling pathways was inhibit and the transcription level of inflammation-related factor Adamts1 was reduced (Amani et al., 2019). Functionalized magnetic Fe_3_O_4_-NPs synthesized with L-carnosine peptide was demonstrated as efficient drug delivery platform for treatment of ischemic stroke, which can cross through the BBB (Lu et al., 2021). The drug loading rate of the NPs was stable at 95.6 ± 0.2% and releasing efficiency of the NPs was controlled and sustainable. Furthermore, the cytotoxicity and the biocompatibility test results were found to be satisfactory. However, inorganic NPs have low biocompatibility due to their inherent toxicity and may not be easily eliminated from the body. What’s worse is that they may cause chronic immune reactions (Yang et al., 2019).

#### Carbon-Based Nanoparticles

Carbon-based NPs (CBNs) are becoming attractive NPs, containing renowned allotropic phases such as amorphous carbon, graphite and diamonds, as well as newly discovered auspicious carbon nanotubes (CNTs), graphene oxide (GO), graphene quantum dots (GQDs) and fullerene (Maiti et al., 2018). Each member of the carbon family exhibits inimitable features and has been widely applied in diverse aspects including drug delivery, imaging, diagnosis, and disease therapy (Bhattacharya et al., 2016). However, for CBNs still contain toxicity, more powerful studies are needed to be focused on the toxicity and pharmacokinetics of CBNs (Maiti et al., 2018). Fuller enol (OH-F) are radical scavengers acting as neuroprotective agents. MRI imaging revealed a significant reduction of infarct volume in ischemic rats treated with OH-F, and these derivatives mitigate the cellular damage and inflammation after stroke. Another study (Sarami Foroshani et al., 2018) has proved that OH-F NPs can reduce the transcription of IL-6 and MMP-9 to protect BBB integrity and relieve brain edema after cerebral ischemia-reperfusion injury.

### Antioxidant Strategies

#### Endogenous Anti-oxidases Enzymes-Loaded Nanoparticles

Several enzymes produced by the human body such as superoxide dismutase (SOD), glutathione peroxidase and catalase; and non-enzymatic natural compounds such as ascorbate, vitamin E, glutathione, thymoquinone and melanin show favorable biocompatibility and robust anti-oxidative properties (Panzella et al., 2013; Couto et al., 2016; Li et al., 2017).

Therapeutic effect based on the native form of SOD are limited because of its short half-life *in vivo* and poor permeability across the BBB (Kinouchi et al., 1991). An approach is based on incorporation of SOD into nano-sized polyion complexes with cationic block copolymers (“nanozymes”). Nanozymes are core-shell structured NPs with the polyion complex core consisting of charge-neutralized polycation chains and protein globules, and the shell consisting of PEG chains. Primary amine groups in the core were cross-linked (*cl*) using low molecular mass chemical cross-linkers to form cl-nanozyme and further purified to improve sample homogeneity by removing non-cl-nanozymes (Manickam et al., 2012). A rat ischemia-reperfusion injury model treated with *cl*-nanozymes-SOD demonstrated a 65% reduction in infarct volume. *cl*-nanozymes-SOD localized in the endothelium of the cerebral vasculature demonstrated a significant reduction in the ROS activity, and protected neurons from undergoing apoptosis (Jiang et al., 2015). Besides, SOD-NPs maintained BBB integrity, thereby relieving cerebral edema. Melanin nanoparticles (MeNPs) is known to function as a potential radical scavenger for more potent and safer antioxidative therapy (Liu et al., 2017). PEG-MeNPs show powerful antioxidative function, and antioxidant targets contains multiple toxic RONS such as O2⋅−, H2O2, ⋅OH, ONOO−, and ⋅NO. In addition to antioxidant function, MeNPs can also inhibit the inflammatory caused by RONS through the suppression of inflammatory mediators and cytokines with negligible side effects.

Another interesting antioxidant compound is Thymoquinone (TQ), which has been proved to possess antioxidant and anti-inflammatory activity. The encapsulation of TQ in mesoporous silica nanocarriers (MSNs) enhanced its delivery to some brain areas (cortex, thalamus, hypothalamus, and midbrain). Furthermore, the activities of SOD and catalase and glutathione level were increased, while the malondialdehyde level was decreased in the brain of MSNs-TQ treated SHRSP rats, indicating its antioxidant (Guan et al., 2018; Fahmy et al., 2019), as shown in Table 2.

**TABLE 2:** Summary of antioxidant drug-loaded nanoparticles and excitotoxicity inhibitors-loaded nanoparticles.

Type of nanoparticles	Nanoparticle	Agent	Mechanism of neuroprotection	Research species	References
**Antioxidant drug-loaded NPs**					
Endogenous anti-oxidases enzymes-loaded NPs	*cl*-nanozymes	SOD	Reduce ROS activity, protect neurons from undergoing apoptosis, and maintain BBB integrity	Animal	Jiang et al., 2015
	PEG-MeNPs	Melanin	Radical scavenger	Animal	Liu et al., 2017
	MSNs-TQ	Thymoquinone	Increase superoxide dismutase and catalase and glutathione level; decrease the malondialdehyde level	Animal	Guan et al., 2018
Exogenous antioxidant drug-loaded NPs	Tempol- TPCD	Tempol	Preserve the tight junctions and suppress neuronal apoptosis, O^2–^ production	Animal	Hosoo et al., 2017; Yuan et al., 2021
	AM	EDV	Eliminate intracellular ROS	Animal	Jin et al., 2017; Hou et al., 2019
	MPP	SCB	Anti-inflammatory and antioxidant; penetrate the BBB more easily	Animal	He et al., 2021b
	SLNs	curcumin	Increase levels of SOD, catalase, glutathione, and mitochondrial complex enzyme activities, decrease levels of the lipid peroxidation, nitrite, and acetylcholinesterase	Animal	Sadegh Malvajerd et al., 2019
**Excitotoxicity inhibitors-NPs**					
Glutamate receptor antagonists-loaded NPs	WGA-NPs	NR2B9c	Protect neurons from NMDA-induced excitotoxicity	Animal	Li et al., 2019
	dual targeted lipid nanomaterials	ZL006	Dissociate nNOS-PSD-95 complex, and then increase dendrite spine density	Animal	Zhao et al., 2016; Lin et al., 2018
Metalloproteinase-1 inhibitor-based NPs	PLGA	TIMP-1	Suppress MMP-9 activity and elevate BBB penetration rate	Animal	Knapska et al., 2013; Zhong et al., 2019

*NPs, nanoparticles; SOD, superoxide dismutase; ROS, reactive oxygen free radicals; PEG, polyethylene glycol; MSNs-TQ, mesoporous silica nanocarriers- Thymoquinone; TPCD, pharmacologically active oligosaccharide material prepared by covalently conjugating a radical-scavenging compound (Tempol) and a hydrogen-peroxide-eliminating moiety of phenylboronic acid pinacol ester (PBAP) on β-cyclodextrin. AM, encapsulated agonistic micelle. MPP/SCB, SCB-loaded pH-sensitive polymeric nanovehicle with a 4T1 cell membrane; MPP, pH-sensitive polymeric nanovehicle with a 4T1 cell membrane. SLNs, solid lipid nanoparticles; WGA-NPs, Wheat germ agglutinin-modified nanoparticle; NMDAR, N-methyl-D-aspartate receptor; PLGA, poly lactic-co-glycolic acid nanoparticles. TIMP-1, tissue inhibitor of matrix metalloproteinases.*

#### Exogenous Antioxidant Drug-Loaded Nanoparticles

Common antioxidant drugs contain Tempol (Hosoo et al., 2017), edaravone (EDV) (Jin et al., 2017), succinobucol (He et al., 2021b), and curcumin (Wang et al., 2019), which were extracted naturally from plants or artificially synthesized. NPs as a feasible strategy extend the half-life of these drugs and improve their ability to cross the BBB, so improve the therapeutic effect of these drugs of ischemic stroke.

In H9c2 cells pre-treated with hypoxia conditions, Tempol could protect H9c2 cells from hypoxia-induced injury through suppressing ROS generation and lipid peroxidation, as well as enhancing antioxidant enzyme activity. Furthermore, the increased expression of Bcl-2 and decreased expression of Bax and caspase-3 in Tempol pre-treatment reduced apoptosis (Jing et al., 2017). During cerebral ischemia-reperfusion injury, pharmacologically active oligosaccharide material prepared by covalently conjugating a radical-scavenging compound (Tempol) and a hydrogen-peroxide-eliminating moiety of phenylboronic acid pinacol ester (PBAP) on β-cyclodextrin (TPCD) preserved the inter-endothelial tight junctions and significantly suppressed neuronal apoptosis, O^2–^ production, thence reduced BBB damage and infarction volume. In general, it can provide neurovascular unit protection (Hosoo et al., 2017; Yuan et al., 2021).

Encapsulated is a clinically approved neuroprotective drug, removing over-produced ROS with an unlimited therapeutic time-window. However, the shortcomings of EDV biological treatment are short circulation half-life and inadequate cerebral uptake. To overcome these weak points, an EDV-encapsulated agonistic micelle (EDV-AM) which adjusted BBB initiatively was developed to specifically deliver EDV into brain ischemia (Jin et al., 2017). The sustainable EDV release of EDV-AM in cells was confirmed by persistent intracellular ROS elimination, providing a basis for EDV-AM to become a promising neuroprotective treatment, especially for ischemic stroke patients who miss the narrow time window of thrombolytic therapy (Hou et al., 2019).

Succinobucol (SCB) (Muldrew and Franks, 2009) is a derivative of probucol, which has lipid-lowering effects with anti-inflammatory and antioxidant properties. SCB has the capacity of preventing the mitochondrial dysfunction induced by tert-butyl hydroperoxide, which provides lines of evidence to clarify SCB as a potential neuroprotective agent (Colle et al., 2013). A SCB-loaded pH-sensitive polymeric nanovehicle with a 4T1 cell membrane (MPP/SCB) (He et al., 2021b), improves the ability to penetrate the BBB and increases the targeting effect of cerebral ischemic lesions. The fluorescence signals of ROS in PC12 cells treated with MPP/SCB was significantly weaker. Meanwhile, the proportion of apoptosis cells was largely reduced, showing remarkable neuroprotective effects in tMCAO rat model.

Another highly concerned anti-inflammatory and antioxidant traditional Chinese medicine component, curcumin (Li et al., 2020), has been delivered by different NPs to overcome its limited stability in circulation. For example, ischemia rats administrated with curcumin loaded solid lipid nanoparticles (C-SLNs) showed increased levels of superoxide dismutase, catalase, glutathione, and mitochondrial complex enzyme activities, while the lipid peroxidation, nitrite, and acetylcholinesterase levels were decreased (Sadegh Malvajerd et al., 2019).

### Excitotoxicity Inhibitors-Loaded Nanoparticles

#### Glutamate Receptor Antagonists-Loaded Nanoparticles

Another line of neuroprotective strategies in cerebral ischemia stroke is the use of neuroprotective compounds to directly target ecotoxicity. Overstimulation of *N*-methyl-D-aspartate receptor (NMDAR) during ischemia-reperfusion stimulates the influx of Ca^2+^ (Wu and Tymianski, 2018). Calcium overload triggers a range of downstream pro-death signals including calpain activation, ROS overload, and mitochondrial damage (Dirnagl et al., 1999), resulting in nerve cell apoptosis. NMDAR channel blockers therapeutic approach may have fewer side effects and/or provide a wider therapeutic window for stroke.

The NR2B9c (Lv et al., 2018) is a well-known peptide confirmed to prevent NMDAR-mediated neurotoxicity without affecting its activity. Wheat germ agglutinin (WGA)-modified NPs carrying NR2B9c (NR2B9c-WGA-NPs) (Li et al., 2019) have a strong ability to cross the BBB for WGA has high affinity for abundant receptors on neuronal surface. NR2B9c-WGA-NPs effectively protected cultured primary cortical neurons from NMDA-induced excitotoxicity, relieved focal ischemic damage in the rat brain, as well as improved their neural function after stroke.

The disruption of the neuronal nitric oxide synthase -postsynaptic density protein-95 (nNOS-PSD-95) can indirectly inhibit the activity of NMDAR, thus preventing excitotoxicity induced by glutamate. ZL006 (Wang et al., 2017) had reformed cerebral ischemic damages and exerted neuroprotective activity in mice and rats subjected to middle cerebral artery occlusion through selectively uncoupling nNOS from PSD-95 induced by ischemia. An animal experiment (Zhao et al., 2016) confirmed that neuroprotectant ZL006 loaded by dual targeted lipid nanomaterials was capable of reducing infarct volume and relieving neurological impairment owing to the NPs could increase drug concentration of ischemic tissue. Neuroplasticity was improved by systemic administration of ZL006 after ischemia as it could reduce excessive neural excitability by dissociating nNOS-PSD-95 complex, and then increase dendrite spine density, thereby improving neuroprotection outcome (Lin et al., 2018).

#### Metalloproteinase-1 Inhibitor-Based Nanoparticles

Matrix metalloproteinases (MMPs) are enzymes capable of cleaving extracellular matrix, membrane, and secreted proteins. The importance of MMPs in ischemic stroke makes it a target for developing novel inhibitors which can serve as promising therapy in patients with stroke (Mashaqi et al., 2021). Among endogenous inhibitor of MMPs, tissue inhibitor of matrix metalloproteinases 1 (TIMP-1) has the greatest therapeutic potential for its strong affinity for MMPs (Sa et al., 2011; Zhong et al., 2019). Generally, the disadvantages of native TIMP-1 include disability to crossing the BBB, short half-life and low bioavailability. However, TIMP-1 wrapped by NPs could increase its bioavailability through enhancing delivery across BBB.

In a hippocampal slice culture model (Chaturvedi et al., 2012), results show that neurons are protected by TIMP-1 from excitotoxicity induced by excitatory amino acid. Further the protection effects are strengthened when TIMP-1 is transported in sustained manner by PLGA NPs. Ps80-coated NPs had an elevated systematic penetration rate, thus Ps80-coated TIMP-1 PLGA NPs may have longer circulation time vivo and better brain permeability compared to PLGA NPs without Ps80-coat. Besides, previous study also showed TIMP-1 was capable of suppressing MMP-9 activity in the brain through direct injection (Knapska et al., 2013).

## Biomimetic Delivery Systems for Neuroprotection Agents

Targeted nanomedicines have shown broad application prospects in the treatment of stroke. However, the disadvantage is the exogenous characteristic with the risk of being cleared by the reticuloendothelial system. To overcome this shortcoming, the bionic nanomedicine delivery strategies containing cells, cell membrane vesicles and exosomes have been widely proposed. Natural NPs found in biological frameworks have been fabricated into new structures that help to build delivery systems by exploit the natural targeting abilities (Sabu et al., 2018). The biomimetic systems find great application in biomedicine, including drug delivery, gene delivery, theranostic, and biosensing applications, owing to their high biocompatibility, less toxicity, and significant interaction.

The mount of the spontaneous cellular regeneration following ischemic stroke is too small to restore the functional neurological. The administration of exogenous stem cells will overcome this limitation and provide neurological restoration (Chan et al., 2017). Stem cell therapeutics as an emerging paradigm for stroke treatment, the safety of which has been overwhelmingly documented, however the efficacy has not been forthcoming (Borlongan, 2019). Several cell types have been studied for immunomodulation in stroke models using intravenous administration and include: mesenchymal stromal cells (MSCs) derived from either the bone-marrow (BM-MSC) (Tan et al., 2018) or adipose-tissues (ADSC) (Gomez-de Frutos et al., 2019), bone-marrow mononuclear cells (BMMNCs) (Li et al., 2016), microglia (Li et al., 2021), or neural stem cells (NSC) (Lee et al., 2008).

Cell membrane coating has recently emerged as a promising biomimetic approach to engineering NPs for targeted drug delivery. By using poly acid NPs wrapped by the membrane of neural stem cells, neuroprotection agents were found an enrichment in ischemic microenvironment, which reduced infarct volumes and improved neurological scores (Ma et al., 2019). A neutrophil-like cell-membrane-coated mesoporous Prussian blue nanozyme (MPBzyme@NCM) (Feng et al., 2021) was delivered into the damaged brain and uptake by microglia to treat the ischemic stroke. The mechanism of ischemic stroke treated by MPBzyme@NCM also included M2 polarization and inhibition of neutrophils recruitment, which significantly relieved inflammatory response. Moreover, MPBzyme@NCM promoted the proliferation of neural stem cells, neuronal precursors, and neurons, that provided a perspective for nanozyme therapy in brain diseases.

Exosomes derived from stem cells with concentrated functional molecules involved in many biological processes and are applied as treatment strategy in cerebral infarct for exosomes showing long-term brain protection through gray matter repair and neurological recovery (Doeppner et al., 2015). Rats treated with exosomes sourced from adipose-derived MSCs (ADSCs) showed improved neurological recovery after brain injury with more remodeling of axons, oligodendrocyte, tract connectivity and myelin (Otero-Ortega et al., 2018). Another animal experiment (Huang et al., 2018) confirmed that exosomes from pigment epithelium-derived factor modified ADSCs ameliorated cerebral ischemic injury by activating autophagy and suppressing neuronal apoptosis.

## Summary and Prospect

Due to the lack of effective treatment strategy, prompt treatment and long-term recovery of stroke patients remain a huge challenge. In decades, nanomedicines have been widely used in the treatment of stroke for it could improve the stability and extend the half-life of drugs *in vivo* (Lu et al., 2019), as well as assist drugs to cross the BBB and realize accumulation at the desired site. Owing to multiple receptors highly expressed on BBB, a steady stream of new targeted nano delivery systems is being developed, which provides new opportunities for the stroke treatment (Bao et al., 2018; Lv et al., 2018). Furthermore, nanoparticle provide possibilities for the realization of emerging therapies, such as gene therapy (Kaviarasi et al., 2019).

It’s difficult to interfere the upstream events of ischemic cascade for their development is very rapid within 1 or 2 h after ischemic attack. Thus, nanomedicines targeting downstream events of ischemic cascade such as oxidative stress, inflammation response, and excitotoxicity deserve more attention in the treatment of stroke. Among numerous biomaterials, liposomes, micelles, and polymeric NPs are mainly investigated for their mature preparation technology and highly likely to be translation into clinical application. In recent years, nanomedicines based on living cells or cell membrane vesicles/exosomes with biocompatibility, safety and targeting properties are becoming research hotspot for targeted treatment of stroke, which bringing new breakthroughs in stroke treatment (Tian et al., 2018; Zhang et al., 2019).

Large number of therapeutic agents targeting a single event in the ischemic stroke were proved may not be effective during clinical trials despite successful inhibition of the specific target. Therefore, combination therapies are more likely to have therapeutic prospects, as the pathophysiology of stroke is complex. Combinations of biomaterial drug-delivery platform with cell transplantation could be a promising direction. The utilization of biomaterials is expected to enhance recovery processes as it could control drug release and achieve a homogenous distribution (Tapeinos et al., 2019). In addition, biomaterials offer structural support and biochemical support to the host tissue, while cell transformation could improve cell survival and tissue regeneration.

## Author Contributions

FF and XW were involved in the study design. KL and LS provided and prepared the materials. JC and JJ wrote the manuscript. All authors contributed to the article and approved the submitted version.

## Conflict of Interest

The authors declare that the research was conducted in the absence of any commercial or financial relationships that could be construed as a potential conflict of interest.

## Publisher’s Note

All claims expressed in this article are solely those of the authors and do not necessarily represent those of their affiliated organizations, or those of the publisher, the editors and the reviewers. Any product that may be evaluated in this article, or claim that may be made by its manufacturer, is not guaranteed or endorsed by the publisher.
